# Clonal Complexity Defines Distinct Tumor‐Intrinsic Biology and Prognosis in Diffuse Large B‐Cell Lymphoma

**DOI:** 10.1002/cam4.71597

**Published:** 2026-02-02

**Authors:** Takahiro Haeno, Kazuko Sakai, Shuji Minamoto, Daiki Nakatsu, Marco A. De Velasco, Shinya Rai, Hirokazu Tanaka, Itaru Matsumura, Kazuto Nishio

**Affiliations:** ^1^ Kindai University Faculty of Medicine Department of Genome Biology Osaka Japan; ^2^ Kindai University Faculty of Medicine Department of Hematology and Rheumatology Osaka Japan; ^3^ Kindai University Center for Genomics, Life Science Research Institute Osaka Japan; ^4^ Centre for Lymphoid Cancer, BC Cancer Vancouver British Columbia Canada

**Keywords:** copy number variations, diffuse large B‐cell lymphoma, DNA mutational analysis, gene expression profiles, genetic heterogeneity

## Abstract

**Background:**

Intratumor heterogeneity (ITH), characterized by the coexistence of genetically distinct subclones within a tumor, has been associated with adverse clinical outcomes in various cancers. However, the clinical and biological implications of ITH in diffuse large B‐cell lymphoma (DLBCL) are still incompletely understood.

**Materials & Methods:**

In this study, we applied a SNP‐array–based approach to assess the clonal complexity in formalin‐fixed, paraffin‐embedded tumor specimens obtained from newly diagnosed patients with advanced‐stage DLBCL (*n* = 74) by calculating the clonal composition (CC) number.

**Rseults:**

Patients with Poly‐CC tumors (CC ≥ 1), which accounted for 79.7% of the cases, had a 5‐year event‐free survival rate of 38.9%, compared with 69.1% in those with Mono‐CC tumors (CC = 0) (Log‐rank p = 0.0520). This association reached statistical significance in the activated B‐cell (ABC) subtype (*n* = 35, Log‐rank *p* = 0.0450) but not in the germinal center B‐cell (GCB) subtype (*n* = 30, Log‐rank p = 0.910). Gene set enrichment analysis revealed upregulation of cell cycle–related pathways in Poly‐CC tumors, consistent with the significantly higher Ki‐67 positivity rate than in Mono‐CC tumors, as confirmed by immunohistochemistry (*p* = 0.00227). Within the ABC subtype, Poly‐CC (Poly‐ABC) tumors exhibited more differentiated transcriptional states and enrichment of IRF4‐associated gene signatures as compared with Mono‐CC (Mono‐ABC) tumors. Conversely, IFN‐γ and IFN‐α response pathways and the IL‐6/JAK‐STAT3 signaling pathway were markedly suppressed in the Poly‐ABC tumors. Furthermore, Poly‐ABC tumors carried a significantly higher number of pathogenic mutations as compared with Mono‐ABC tumors (*p* = 0.0147).

**Conclusion:**

These results suggest that clonal complexity captures tumor‐intrinsic features and biological diversity in DLBCL, especially in the ABC subtype, offering novel insights into the disease pathogenesis.

## Graphical abstract

High intratumor heterogeneity in DLBCL is associated with reduced event‐free survival, particularly in the ABC subtype. Tumors with high clonal composition exhibit more aggressive biological behavior compared to monoclonal tumors in the ABC subtype of DLBCL.

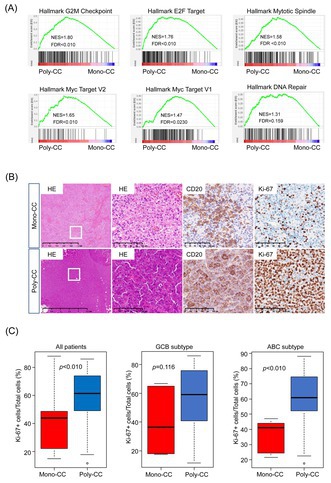

**Abbreviations**
ABC: activated B‐cell‐like
BAF: B‐allele frequency
CC: clonal composition
CHOP: cyclophosphamide‐doxorubicin‐vincristine‐prednisolone
CNVs: copy number variations
COO: cell‐of‐origin
CVP: cyclophosphamide‐vincristine‐prednisolone
DLBCL: diffuse large B‐cell lymphoma
EBV: Epstein–Barr virus
EFS: event free survival
FFPE: formalin‐fixed, paraffin‐embedded
GCB: germinal center B‐cell‐like
GSEA: Gene set enrichment analysis
HCC: hepatocellular carcinoma
IFN: interferon
IL: interleukin
IRF4: interferon regulatory factor 4
ITH: intratumor heterogeneity
LDH: lactate dehydrogenase
Log2R: log2 ratio
MATH: mutant‐allele tumor heterogeneity
MIP: molecular inversion probe
MSigDB: Molecular Signatures Database
OS: overall survival
R: rituximab
SNP: single nucleotide polymorphism
ssGSEA: single‐sample GSEA
STAT: signal transducer and activator of transcription
THPCOP: pirarubicin‐cyclophosphamide‐vincristine‐prednisolone
TME: tumor microenvironment
TPM: transcripts per million
WHO: world health organization

## 1 Introduction

Intratumor heterogeneity (ITH) refers to the presence of genetically and phenotypically distinct subclones within a tumor, and among lesions in the same patient [1]. This heterogeneity arises through a combination of tumor‐intrinsic factors, such as genomic alterations and epigenetic modifications, and extrinsic factors, including regional variations in the tumor microenvironment [1, 2, 3]. ITH enables clonal selection under therapeutic pressure, whereby resistant subclones persist and contribute to treatment failure and relapse [1]. Several studies in solid tumors have shown that high levels of ITH are associated with inferior clinical outcomes [1, 4, 5, 6, 7, 8, 9, 10].

Diffuse large B‐cell lymphoma (DLBCL) is the most common subtype of non‐Hodgkin lymphoma, accounting for 30%–40% of all cases [11], and is a clinically and biologically heterogeneous disease. Despite the tumor heterogeneity, frontline treatment remains largely uniform, typically consisting of the R‐CHOP regimen (rituximab, cyclophosphamide, doxorubicin, vincristine, and prednisolone). Although over 60% of patients achieve cure with this regimen, a substantial proportion of patients experiences relapse or refractory disease [12, 13, 14]. Accordingly, classification strategies have been developed to capture this inter‐patient tumor heterogeneity, with the goal of guiding future treatment stratification. These efforts began with gene expression profiling (or cell‐of‐origin)–based classification of DLBCL into the germinal center B‐cell‐like (GCB) and activated B‐cell‐like (ABC) subtypes [15, 16]. More recently, genetic classifications based on recurrent mutations and structural alterations have further refined this approach [17, 18, 19, 20, 21, 22]. However, these frameworks still fall short of fully capturing the biological and clinical diversity of DLBCL. In particular, ITH has been investigated in only a limited number of studies [23, 24] representing a critical gap in our understanding of this biologically complex lymphoma.

Genomic structural changes such as gene amplifications or deletions arise from copy number variations (CNVs), which can be detected using array‐based technologies such as chromosomal microarrays [25, 26]. The OncoScan formalin‐fixed, paraffin‐embedded (FFPE) assay utilizes the molecular inversion probe (MIP) technology to measure genome‐wide CNVs and loss of heterozygosity, providing the B‐allele frequency (BAF), log2 ratio (log2R), and copy number at over 220,000 polymorphic genomic loci [27]. To quantify ITH based on the CNVs data, we developed the Clonal Composition (CC) number, a metric derived from the BAF and log2R values that reflects the number of coexisting subclones within a tumor. Using this approach, we previously analyzed tumor specimens of ovarian cancer and hepatocellular carcinoma (HCC), and found that Poly‐CC tumors, characterized by multiple subclonal populations, were associated with poorer clinical outcomes as compared with Mono‐CC tumors [28, 29]. These observations prompted us to extend our approach beyond solid tumors to explore its potential in DLBCL.

In this study, we applied our CC number–based approach to patients with newly diagnosed, advanced‐stage DLBCL to quantify the clonal diversity within a tumor and evaluate its association with the gene expression profiles, mutational landscapes, tumor microenvironment, and clinical outcomes to better understand how clonal diversity impacts disease biology.

## 2 Materials and Methods

### 2.1 Study Patients and Clinical Data

We enrolled 86 patients who were newly diagnosed as having DLBCL according to the WHO classification 2008 and treated at Kindai University Hospital between January 2016 and March 2023. We excluded cases of indolent lymphomas that had transformed to DLBCL and those with primary central nervous system involvement, but included cases of systemic lymphoma with central nervous system involvement. This study was conducted with the approval of the independent ethics committee of Kindai University Faculty of Medicine (R03‐283), adhering to the principles of the Declaration of Helsinki using an opt‐out method.

### 2.2 Clonal Composition Analysis

To analyze the clonal composition, a whole‐genome copy number assay was used with the OncoScan FFPE assay (Thermo Fisher Scientific). Clonal composition numbers were calculated using the BAF and log2R values obtained from the OncoScan FFPE assay using the Onco Clone Composition program as previously described [28, 29], and are described in detail in the Methods S1 section.

### 2.3 RNA Sequencing

Total RNA from each sample was prepared for sequencing using the SMART‐Seq Stranded Kit (Takara Bio). The libraries were sequenced on Illumina NovaSeq X Plus (Illumina). Raw data were processed by the DRAGEN Bio‐IT Platform (version 3.7.5, Illumina), and transcripts per million (TPM) were utilized for normalization. The cell of origin was determined based on the results of gene expression profiling described in previous reports [30]. The ecotype of each DLBCL was predicted using the Lymphoma ECOtyper [31]. Analysis of tumor microenvironment was performed using the CIBERSORTx [32], and is described in detail in Methods S1 section.

### 2.4 Target DNA Sequencing

Target DNA sequencing was performed with an Oncomine Lymphoma Panel (Thermo Fisher Scientific) and is described in detail in the Supplementary Methods section.

### 2.5 Gene Set Enrichment Analysis

Gene set enrichment analysis (GSEA) was performed to identify pathways enriched in the Mono‐CC tumors and Poly‐CC tumors using the Molecular Signature Database (MSigDB) Hallmark gene sets. A nominal *p* value of < 0.05 and a false discovery rate *q* value of < 0.25 were considered as indicative of statistical significance. Single‐sample GSEA (ssGSEA) was performed using Signature DB to compare the gene expression signatures score between Mono‐CC tumors and Poly‐CC tumors [33, 34].

### 2.6 Immunohistochemical Staining

Immunohistochemical staining was performed using the automated Ventana the BenchMark Ultra platform (Ventana Medical System) and is described in detail in the Supplementary Methods section.

### 2.7 Statistical Analysis

The event free survival (EFS) was defined as the length of time from the day of diagnosis to the day of diagnosis of progression, day of discontinuation of treatment, or death from any cause. Overall survival (OS) was defined as the length of time from the day of diagnosis to death from any cause. The EFS and OS were calculated by the Kaplan–Meier method and compared by the log‐rank test. Categorical data were analyzed by Fisher's exact test or the Kruskal–Wallis test. Univariate analyses were performed using a Cox proportional hazards model. All the statistical analyses were performed using EZR (Saitama Medical Center, Jichi Medical University) [35] and *p* values of < 0.05 were considered as statistical significance.

Based on the observed allocation (Mono‐CC: *n* = 15, Poly‐CC: *n* = 59) and event rates, we calculated that, at 80% power and a two‐sided α = 0.05, the minimum detectable hazard ratio was approximately 2.3. The observed hazard ratio for event‐free survival in the overall cohort was 1.9 (95% CI: 0.98–3.74), indicating that the study was slightly underpowered to detect effects of this magnitude.

## 3 Results

### 3.1 Clonal Composition of Newly Diagnosed DLBCL

CC number reflects the estimated number of clones in the tumor tissue. We established a method to estimate the CC number in FFPE tumor specimens using a whole‐genome SNP array and the Onco Clone Composition estimation program [28]. The flowchart of the analysis is shown in Figure S1. Using our approach, we could estimate the tumor CC number in 74 of the 86 patients (86.0%) initially enrolled in our study. CC number calculation failed in the remaining 12 patients (14.0%) due to inadequate DNA quality. Representative examples of tumors with different clonal composition (CC = 1, 2, and 3) are shown in Figure S2A–C. In contrast to our prior ovarian cancer study, we defined patients with CC = 0 as having no detectable intra‐tumor heterogeneity (Mono‐CC tumors), whereas patients with CC ≥ 1 were classified as exhibiting intra‐tumor heterogeneity (Poly‐CC tumors). We adopted CC = 0 as the criterion for Mono‐CC to ensure that “monoclonal” strictly reflects the absence of any detectable subclonality in this dataset. This decision was based on the observation that several CC = 1 tumors exhibit small, coherent BAF/log2R deviations across contiguous segments that fall below the 1% clone‐calling footprint; when the footprint is adjusted to 0.5%, these segments form a second cluster (i.e., CC = 1 becomes CC = 2). Therefore, using CC = 0 as Mono‐CC provides a stricter and biologically meaningful definition in this dataset. Figure S2D–E shows representative profiles of two CC = 1 cases showing small but reproducible segments. Table 1 summarizes the clinical characteristics of all the 74 patients. The median age was 73 years (range 24–92), and 47% of the patients were male. All patients had advanced clinical disease (stage III, *n* = 20; stage IV, *n* = 54) and received rituximab‐based chemotherapy as first‐line therapy (R‐CHOP, *n* = 52; R‐THPCOP [pirarubicin, cyclophosphamide, vincristine, prednisolone], *n* = 21; R‐CVP [cyclophosphamide, vincristine, prednisolone], *n* = 1). Three of the patients in the study cohort were diagnosed as having Epstein–Barr virus (EBV)‐positive DLBCL. The cell‐of‐origin (COO) was determined based on gene expression profiling, performed in accordance with procedures described in previous reports [30]. Of the 74 patients with DLBCL, 30 patients (40.5%) had the GCB subtype, 35 patients (47.3%) had the ABC subtype, and 8 patients (10.8%) had an Unclassified subtype. The COO could not be determined in one patient.

**TABLE 1:** Characteristics of DLBCL patients with Mono‐CC and Poly‐CC tumors.

	Total (*n* = 74)	Mono‐CC (*n* = 15)	Poly‐CC (*n* = 59)	*p* value
Clonal composition number, *n* (%)				
Mean (SD)	1.01 (0.72)	0 (0)	1.27 (0.57)	—
Median (range)	1 (0–4)	0 (–)	1 (1–4)	
0	15 (21)	15 (100)	0 (0)	
1	46 (62)	0 (0)	46 (78)	
2	11 (15)	0 (0)	11 (18)	
3	1 (1)	0 (0)	1 (2)	
4	1 (1)	0 (0)	1 (2)	
Age at diagnosis, years				
Median (range)	73 (24–92)	73 (24–85)	72 (26–92)	1.00
< 61, *n* (%)	8 (11)	1 (7)	7 (12)	
≥ 61, *n* (%)	66 (89)	14 (93)	52 (88)	
Sex, *n* (%)				
Male	35 (47)	7 (47)	28 (47)	1.00
Female	39 (53)	8 (53)	31 (53)	
Cell of origin, *n* (%)				
GCB	30 (41)	6 (40)	24 (41)	0.914
ABC	35 (47)	6 (40)	29 (49)	
Unclassified	8 (11)	2 (13)	6 (10)	
NA	1 (1)	1 (7)	0 (0)	
1st therapy, *n* (%)				
R‐CHOP	52 (70)	11 (73)	41 (69)	0.192
R‐THPCOP	21 (28)	3 (20)	18 (31)	
R‐CVP	1 (2)	1 (7)	0 (0)	
Stage, *n* (%)				
III	20 (27)	4 (27)	16 (27)	1.00
IV	54 (73)	11 (73)	43 (73)	
LDH (U/L)				
Median	306 (140–3726)	282 (191–1705)	318 (140–3726)	1.00
Low	19 (26)	4 (27)	15 (25)	
High	55 (74)	11 (73)	44 (75)	
Number of extranodal sites, *n* (%)				
0–1	40 (54)	9 (60)	31 (53)	0.773
≥ 2	34 (46)	6 (40)	28 (47)	
ECOG PS, *n* (%)				
0–1	63 (85)	14 (93)	49 (83)	0.443
2–4	11 (15)	1 (7)	10 (17)	
IPI, *n* (%)				
Low‐int low	16 (22)	3 (20)	13 (22)	1.00
Int high‐high	58 (78)	12 (80)	46 (78)	
EBV‐DLBCL, *n* (%)				
Yes	3 (4)	1 (7)	2 (3)	0.499
No	71 (96)	14 (93)	57 (97)	

Abbreviations: ABC, Activated B cell subtype; CC, Clonal composition; EBV, Epstein–Barr virus; GCB, Germinal center subtype; IPI, International prognostic index; LDH, Lactate dehydrogenase; NA, not available; R‐CHOP, Rituximab‐cyclophosphamide, doxorubicin, vincristine, prednisolone; R‐CVP, Rituximab‐cyclophosphamide, vincristine, prednisolone; R‐THPCOP, Rituximab‐pirarubicin, cyclophosphamide, vincristine, prednisolone.

Based on the results of the CC number, 15 patients (20.3%) were classified as having Mono‐CC DLBCL (Mono‐CC group) and 59 patients (79.7%) as having Poly‐CC DLBCL (Poly‐CC group: CC = 1, *n* = 46; CC = 2, *n* = 11; CC = 3, *n* = 1; CC = 4, *n* = 1). Table 1 shows the patient characteristics according to the CC number. There was no statistically significant difference in the age, clinical disease stage, serum lactate dehydrogenase (LDH) level, number of extranodal disease sites, or performance status between the patients with Mono‐CC tumors and Poly‐CC tumors. The patient characteristics according to the CC number within each COO subtype are shown in Table 2. There were no significant differences in the clinical background characteristics between the patients with Mono‐CC tumors and Poly‐CC tumors within any of the COO subtypes.

**TABLE 2:** Characteristics of DLBCL patients classified according to the cell‐of‐origin (COO) classification (GCB, ABC, and Unclassified) with Mono‐CC and Poly‐CC tumors.

	GCB subtype		*p* value	ABC subtype		*p* value
	Mono‐CC (*n* = 6)	Poly‐CC (*n* = 24)		Mono‐CC (*n* = 6)	Poly‐CC (*n* = 29)	
Clonal composition number, *n* (%)						
Mean (SD)	0 (0)	1.16 (0.37)	—	0 (0)	1.34 (0.65)	—
Median (range)	0 (–)	1 (1–2)		0 (–)	1 (1–4)	
0	6 (100)	0 (0)		6 (100)	0 (0)	
1	0 (0)	20 (83)		0 (0)	21 (72)	
2	0 (0)	4 (17)		0 (0)	7 (24)	
3	0 (0)	0 (0)		0 (0)	0 (0)	
4	0 (0)	0 (0)		0 (0)	1 (4)	
Age at diagnosis, years						
Median (range)	74 (65–83)	72 (26–92)	0.557	71 (67–85)	75 (48–87)	1.00
< 61, *n* (%)	0 (0)	4 (17)		0 (0)	1 (3)	
≥ 61, *n* (%)	6 (100)	20 (83)		6 (100)	28 (97)	
Sex, *n* (%)						
Male	5 (83)	11 (46)	0.175	1 (17)	13 (45)	0.366
Female	1 (17)	13 (54)		5 (83)	16 (55)	
1st therapy, *n* (%)						
R‐CHOP	5 (83)	18 (75)	1.00	4 (66)	17 (59)	0.159
R‐THPCOP	1 (17)	6 (25)		1 (17)	12 (41)	
R‐CVP	0 (0)	0 (0)		1 (17)	0 (0)	
Stage, *n* (%)						
III	1 (17)	8 (33)	0.637	2 (33)	5 (17)	0.576
IV	5 (83)	16 (67)		4 (67)	24 (83)	
LDH (U/L)						
Median (range)	283 (208–1705)	311 (191–1719)	0.603	286 (191–800)	346 (154–3726)	1.00
Low	2 (33)	5 (21)		2 (33)	9 (31)	
High	4 (67)	19 (79)		4 (67)	20 (69)	
Number of extranodal sites, *n* (%)						
0–1	3 (50)	12 (50)	1.00	5 (83)	15 (52)	0.207
≥ 2	3 (50)	12 (50)		1 (17)	14 (48)	
ECOG PS, *n* (%)						
0–1	6 (100)	22 (92)	1.00	5 (83)	22 (76)	1.00
2–4	0 (0)	2 (8)		1 (17)	7 (24)	
IPI, *n* (%)						
Low‐int low	2 (33)	5 (21)	0.603	1 (17)	6 (21)	1.00
Int high‐high	4 (67)	19 (79)		5 (83)	23 (79)	
EBV‐DLBCL, *n* (%)						
Yes	0 (0)	0 (0)	—	1 (17)	1 (3)	0.318
No	6 (100)	24 (100)		5 (83)	28 (97)	

Abbreviations: ABC, Activated B cell subtype; CC, Clonal composition; EBV, Epstein–Barr virus; GCB, Germinal center subtype; IPI, International prognostic index; LDH, Lactate dehydrogenase; R‐CHOP, Rituximab‐cyclophosphamide, doxorubicin, vincristine, prednisolone; R‐CVP, Rituximab‐cyclophosphamide, vincristine, prednisolone; R‐THPCOP, Rituximab‐pirarubicin, cyclophosphamide, vincristine, prednisolone.

### 3.2 Clinical Outcomes

**FIGURE 1:**
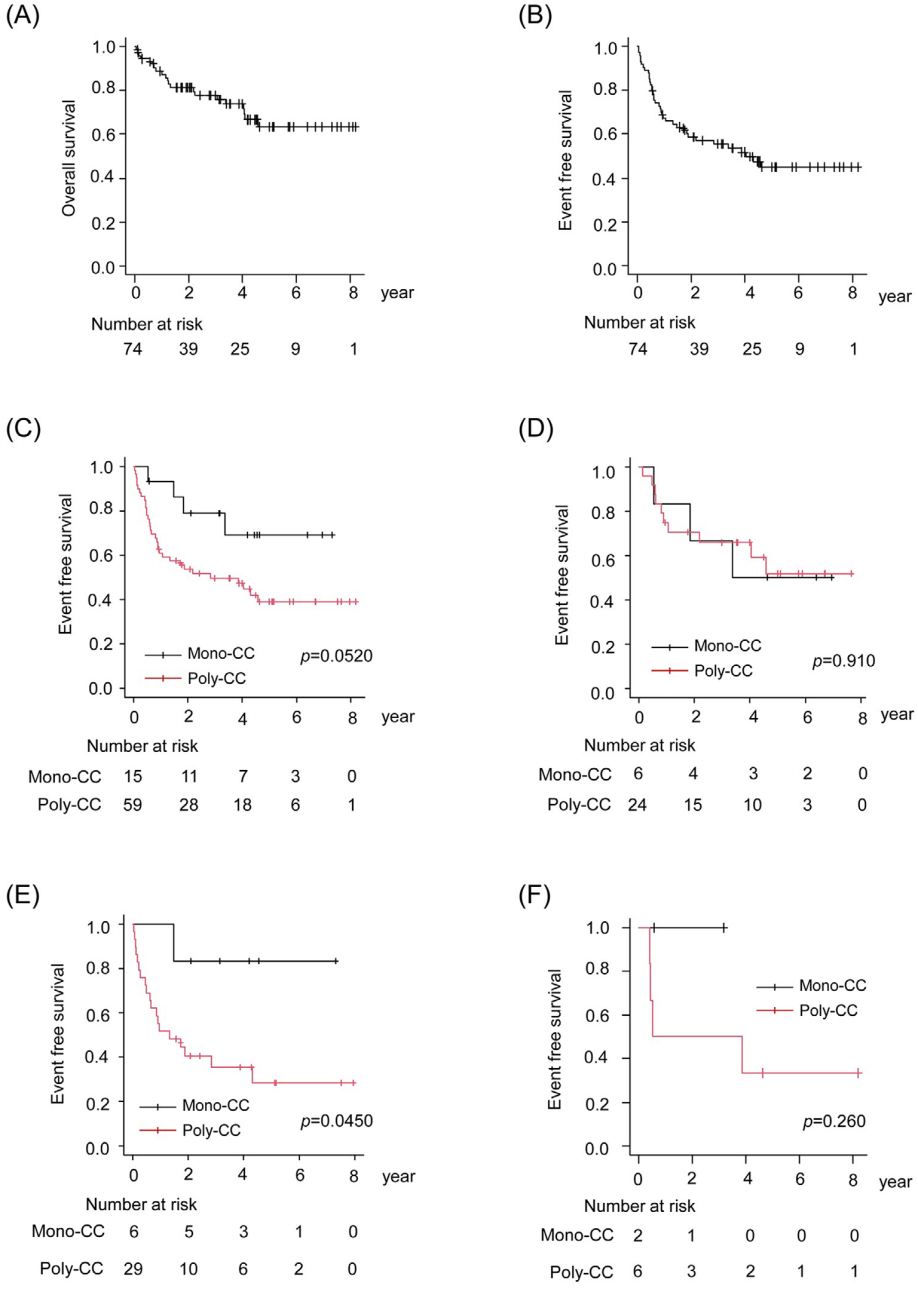
Differences in the event‐free survival (EFS) and overall survival (OS) in patients with diffuse large B‐cell lymphoma (DLBCL) based on the clonal composition. (A) Kaplan–Meier curves for OS for all patients (*n* = 74). (B) Kaplan–Meier curves for EFS for all patients (*n* = 74). (C) Kaplan–Meier curves for EFS based on the clonal composition. (D) Kaplan–Meier curves for EFS based on the clonal composition in patients with GCB‐DLBCL. (E) Kaplan–Meier curves for EFS based on the clonal composition in patients with ABC‐DLBCL. (F) Kaplan–Meier curves for EFS based on the clonal composition in patients with Unclassified‐DLBCL; the *p* values listed on each graph were calculated by the log‐rank test.

At a median follow‐up time of 1196 days (range, 17–2989 days), the 5‐year OS and EFS rates were 63.3% (95% CI, 48.3%–75.1%) and 44.8% (95% CI, 31.9%–56.9%), respectively, in the 74 patients for whom the CC number could be calculated (Figure 1A,B). The 5‐year EFS rate was 37.8% (95% CI, 19.9%–55.6%) in DLBCL patients with the ABC subtype, and 52.0% (95% CI, 31.2%–69.3%) in patients with the GCB subtype, respectively (Figure S3A), with no statistically significant difference (Log‐rank *p* = 0.13, HR: 1.71, 95% CI, 0.84–3.45). Similarly, no significant difference in OS was observed between the ABC and GCB groups (Figure S3B).

The 5‐year EFS rate was 69.1% (95% CI, 36.1%–87.5%) in patients with Mono‐CC tumors and 38.9% (95% CI, 25.2%–52.4%), respectively (Figure 1C), with no statistically significant difference (Log‐rank *p* = 0.0520, HR: 2.68, 95% CI, 0.94–7.57). The 5‐year OS rates were comparable between the two patient groups (Mono‐CC group: 66.2%; 95% CI, 32.4%–86.0%; Poly‐CC group: 62.8%; 95% CI, 45.6%–75.9%, HR: 1.27, 95% CI, 0.42–3.78) (Figure S3C).

Integrating the results for the COO and CC number, we found that the 5‐year EFS rate was 50.0% (95% CI, 11.1%–80.4%) in the Mono‐CC GCB group (*n* = 6), and 51.9% (95% CI, 27.5%–71.6%) in the Poly‐CC GCB group (*n* = 24), with no statistically significant difference between the two patient groups (Log‐rank *p* = 0.910, HR: 0.92, 95% CI, 0.25–3.38) (Figure 1D). However, among the DLBCL patients with the ABC subtype, the 5‐year EFS rate was significantly lower in the Poly‐CC ABC group (28.4%; 95% CI: 11.5%–48.0%) as compared with that in the Mono‐CC ABC group (83.3%; 95% CI: 27.3%–97.5%) (Log‐rank *p* = 0.0450, HR: 6.08, 95% CI, 0.81–45.6) (Figure 1E), suggesting that clonal complexity may be associated with a poorer prognosis especially in DLBCL patients with the ABC subtype.

Although Poly‐CC tumors were more frequently treated with R‐THPCOP and exhibited more extensive extranodal involvement, multivariable analysis adjusting for these factors did not abolish the adverse trend associated with Poly‐CC (Table S2). Within the ABC subtype, both regimen and extranodal site count also showed borderline associations in the same adverse direction (Table S3).

### 3.3 Differences in the Tumor Cell Proliferative Activity Between Mono‐CC and Poly‐CC DLBCL

**FIGURE 2:**
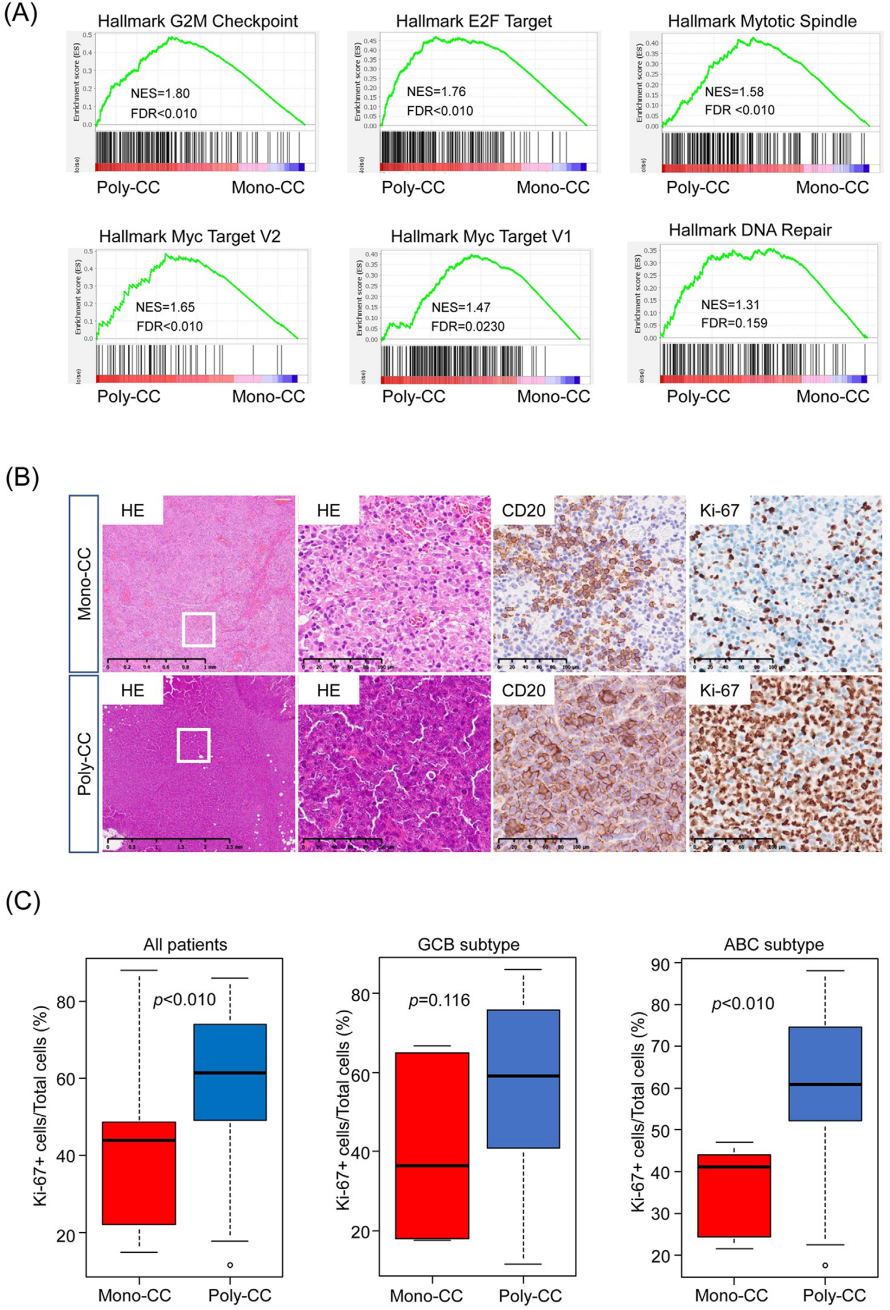
Gene set enrichment analysis (GSEA) and immunohistochemical analysis in patients with Mono‐CC and Poly‐CC. (A) Gene set enrichment plot of positive enrichment in Poly‐CC DLBCL as compared with Mono‐CC DLBCL. The normalized enrichment score (NES) and false‐discovery rate (FDR) values are indicated in all the GSEA plots. (B) Representative HE staining and immunohistochemical (IHC) staining of Mono‐CC (upper) and Poly‐CC (lower) tumor specimens. Shown from left are HE staining (×2.5 magnification), HE staining (×20 magnification), IHC staining for CD20 (×20 magnification), and IHC staining for Ki‐67 (×20 magnification). (C) Quantitative analysis of Ki‐67 as the number of Ki‐67 positive cells/total cell number in mono‐CC and poly‐CC tumors. Box plots shown are for the entire group (left), GCB‐DLBCL (middle), and ABC‐DLBCL (right). HE, hematoxylin–eosin.

Given the trend toward a poorer EFS in the Poly‐CC group observed in the overall cohort, we first investigated the underlying biological tumor characteristics in all the 74 patients for whom gene expression data were available. We performed gene set enrichment analysis (GSEA) using bulk gene expression data and gene sets from the MSigDB database. GSEA showed that gene sets related to cell proliferation and the cell cycle, including the G2M checkpoint, E2F targets, MYC targets, mitotic spindles, and DNA repair, were significantly upregulated, suggestive of enhanced tumor cell proliferative activity in the Poly‐CC tumors (Figure 2A; nominal *p* < 0.05, FDR *q* < 0.25).

To validate these findings at the protein level, we performed immunohistochemical staining for Ki‐67, a widely used proliferation marker in DLBCL, to assess cell cycle dysregulation. In line with the findings of GSEA, the proportion of Ki‐67^+^ tumor cells was significantly higher in the Poly‐CC group than in the Mono‐CC group across all samples, including both tumors of the ABC and GCB subtypes (Figure 2C left, *p* = 0.00227). This association remained significant within the ABC subtypes (Figure 2C right, *p* = 0.00152) but not in the GCB subtype (Figure 2C middle, *p* = 0.116). These results confirmed increased tumor cell proliferative activity in the Poly‐CC group, particularly in DLBCL patients with the ABC subtype, which aligns with the poorer EFS observed in this subgroup.

To further explore potential quantitative relationships between CC number and biological or clinical features such as Ki‐67 index, IPI and clinical stage, we performed Pearson's correlation analysis and Mann–Whitney U test. Figure S4A–C shows a positive association between CC number and Ki‐67 index in the overall cohort (*r* = 0.273, *p* = 0.0184) (Figure S4A), although the correlations were modest. This relationship was more pronounced in the ABC subtype (*r* = 0.387, *p* = 0.0216) (Figure S4C), whereas no significant correlation was observed in the GCB subtype (*r* = 0.119, *p* = 0.531) (Figure S4B). However, no significant correlation was observed between CC number and IPI or clinical stage (Figure S4D–I). These findings suggest that clonal complexity may be more closely linked to proliferative activity in ABC‐type DLBCL, highlighting potential biological differences across subtypes.

### 3.4 Differential Malignant B‐Cell States Between Mono‐CC and Poly‐CC DLBCL

**FIGURE 3:**
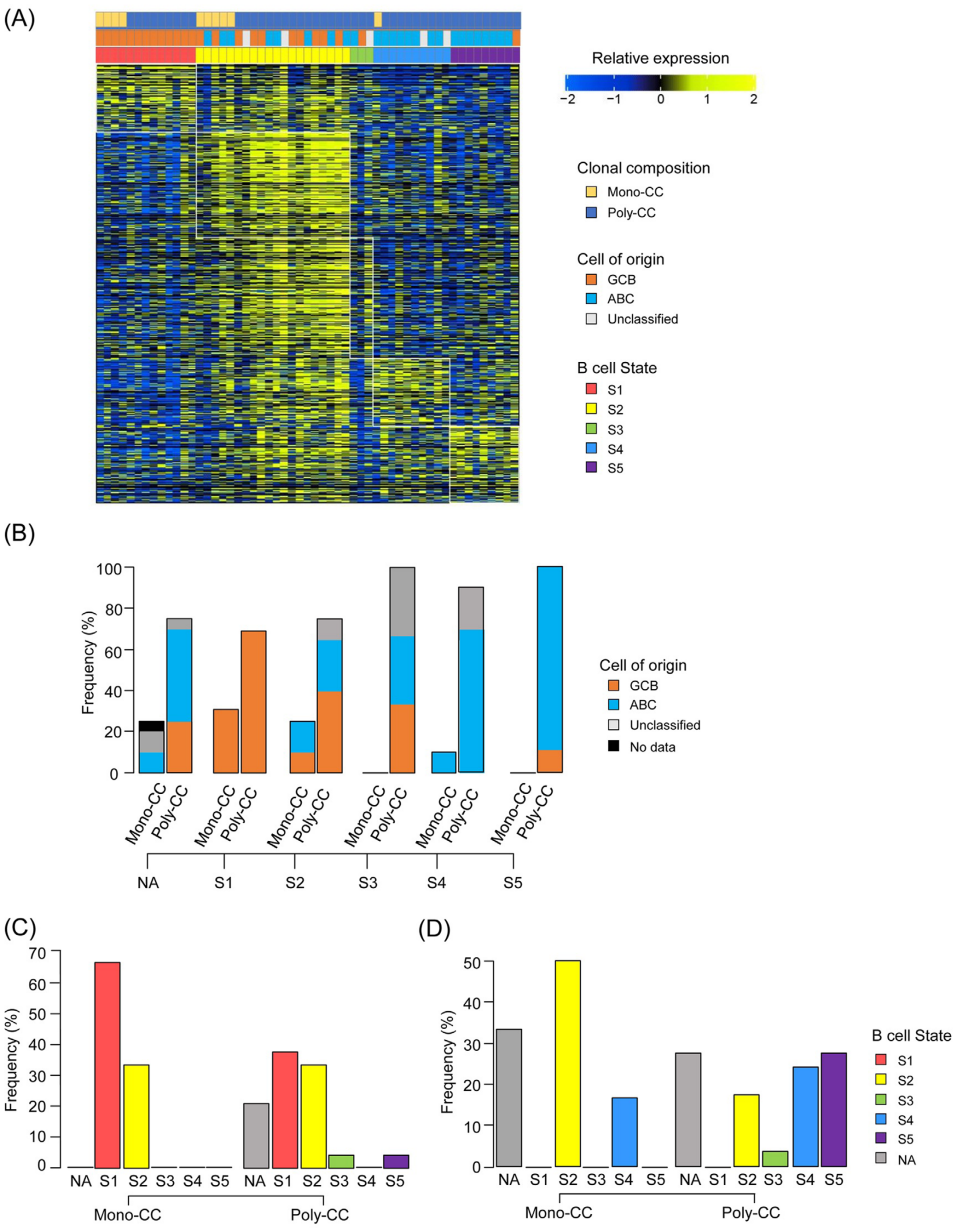
Assessment of B cell status in DLBCL using ECOtyper. (A) Heatmap of the tumor cell ecotypes based on different clonal compositions. The states of B cells from S1 to S5 indicate malignancy, with a canonical marker gene from GCB‐DLBCL, whereas states S4 and S5 express marker genes of ABC‐DLBCL. (B) The frequency distribution of CC numbers in each tumor B cell state. The COO classification is color‐coded in the bar graph. (C) The frequency of tumor B cell states for each CC number in DLBCL patients with the GCB subtype. (D) The frequency of tumor B cell states for each CC number in DLBCL patients with the ABC subtype. NA, not assigned.

Next, to investigate differences in the differentiation states of the malignant B cells according to CC number, we applied a previously reported tool, Lymphoma ECOtyper [31]. Using this tool, we successfully assigned B‐cell states (S1–S5) to the tumor cells in 55 of the 74 patients, while being unable to categorize the cells in the remaining 19 patients (Figure 3A). Among the 55 patients for whom both the B‐cell state and CC classification were available, S1 (*n* = 13) was exclusively associated with the GCB subtype (13/13), whereas S4 (*n* = 10) and S5 (*n* = 9) were predominantly associated with the ABC subtype (8/10 and 8/9, respectively) (Figure 3B), in agreement with previous reports on the relationship between the COO subtypes and B‐cell maturation states [31].

Among the patients with the GCB subtype, analysis using the ECOtyper tool revealed earlier B cell states in all of the patients with Mono‐CC GCB (S1, 66.7%; S2, 33.3%, Figure 3C); similarly, the analysis also revealed earlier B cell states in the majority of patients with Poly‐CC GCB (S1, 37.5%; S2, 33.3% Figure 3C). Among patients with the ABC subtype, ECOtyper classified the B cell state as S2 in half of the patients with Mono‐CC ABC (S2, 50.0%, Figure 3D), while it classified the B cell state into the more differentiated states of S4 or S5, known to be derived from differentiated plasma‐like cells, in a little over half of the patients with Poly‐CC ABC (S4–S5, 51.7%, Figure 3D). These results suggest that the B‐cell differentiation state is skewed toward a later stage of differentiation in Poly‐CC DLBCL as compared with Mono‐CC DLBCL, particularly within the ABC subtype.

ECOtyper assigned lymphoma ecotypes (Figure S5, LE1–LE9) across the cohort. LE1, LE2, and LE6 were observed exclusively among poly‐CC tumors, whereas LE4 appeared only among mono‐CC tumors. Given the small numbers within individual ecotypes, these patterns should be considered descriptive; no significant association between ecotype and clonal complexity was detected.

**FIGURE 4:**
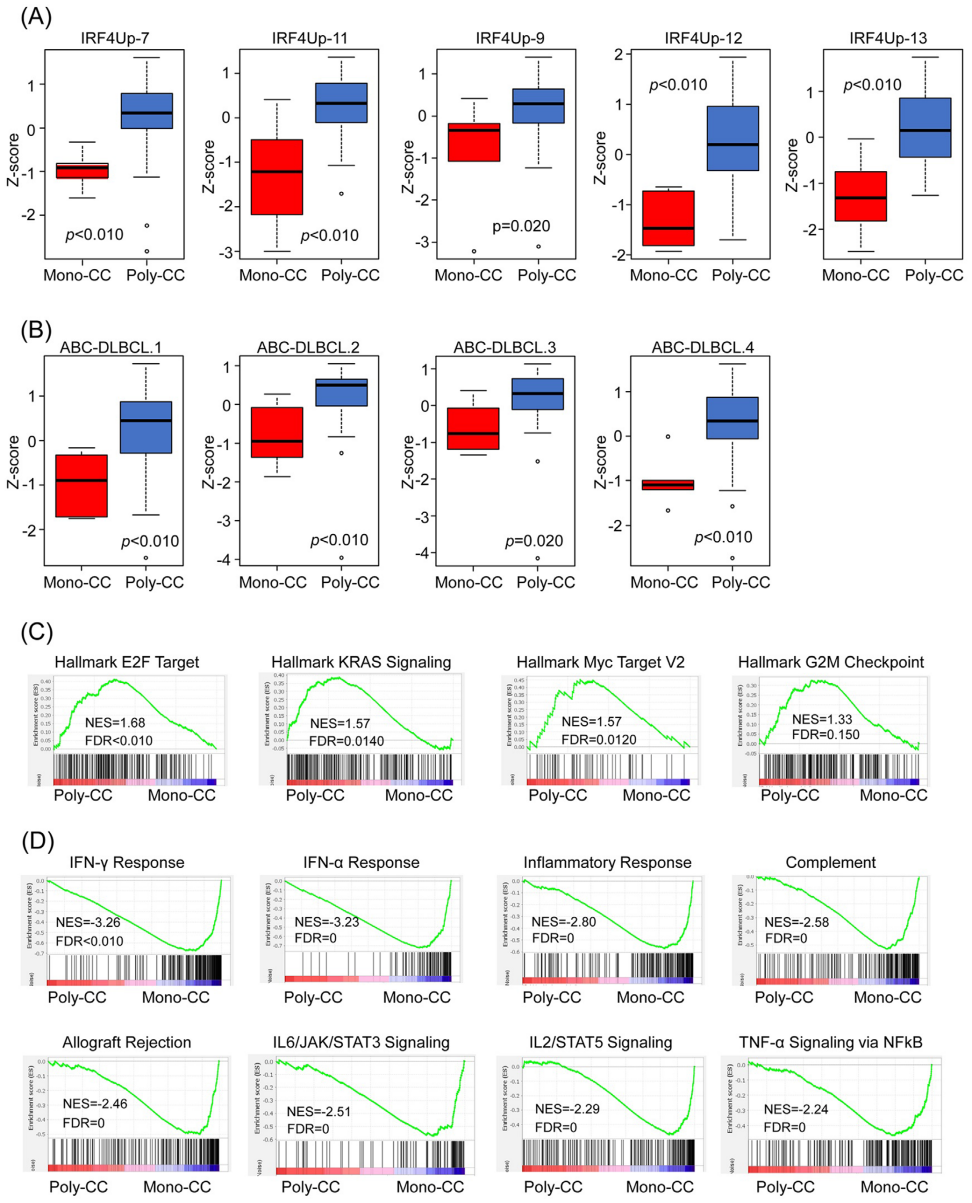
Distinct biological behaviors of Mono‐CC and Poly‐CC tumors within the ABC subtype of DLBCL. Differential expressions of *IRF4* and ABC‐DLBCL‐specific gene sets were calculated using ssGSEA. Signature scores shown in the box plots were normalized by the *Z*‐score method. *p* values were calculated using the Mann–Whitney U test. (A) Comparison of the gene expression signatures of *IRF4*‐specific gene sets between Mono‐CC and Poly‐CC tumors in DLBCL patients with the ABC subtype. (B) Comparison of the gene expression signatures of ABC‐DLBCL‐specific gene sets between Mono‐CC and Poly‐CC tumors in DLBCL patients with the ABC subtype. GSEA was used to compare the gene expression profiles of Mono‐CC and Poly‐CC tumors in DLBCL patients with the ABC subtype. (C) Upregulated hallmark signatures in Poly‐CC ABC DLBCL as compared with Mono‐CC ABC DLBCL. (D) Downregulated hallmark signatures in Poly‐CC ABC DLBCL as compared with Mono‐CC ABC DLBCL. The values of the normalized enrichment score (NES) and FDR (false‐discovery rate) are indicated in all the GSEA plots.

To validate the ECOtyper‐based inference, we performed both GSEA and single‐sample GSEA (ssGSEA), which allows pathway activity to be quantified at the individual sample level [33, 34]. Since the differences in B‐cell differentiation states between Mono‐CC and Poly‐CC tumors were particularly pronounced in the ABC subtype of DLBCL, we focused our analysis on this group. A total of nine gene sets, including 5 molecular signatures associated with upregulation of *IRF4* (IRF4Up‐7, IRF4Up‐9, IRF4Up‐11, IRF4Up‐12, IRF4Up‐13), a transcription factor critical for B‐cell differentiation‐specific gene sets [36, 37, 38], and ABC‐DLBCL (ABCDLBC‐1, ABCDLBCL‐2, ABCDLBC‐3, ABCDLBC‐4)‐specific gene sets, were included. Gene sets are derived from the gene expression classifier found in SignatureDB (https://lymphochip.nih.gov/signaturedb/index.html). Using ssGSEA, we first confirmed that the gene signatures associated with *IRF4* were significantly enriched in the Poly‐CC ABC tumors as compared with the Mono‐CC ABC tumors (Figure 4A). In addition, the expressions of ABC‐DLBCL‐specific gene signatures were also upregulated in the Poly‐CC ABC tumors (Figure 4B).

We then performed GSEA to further explore the functional differences between the Mono‐CC and Poly‐CC tumors within the ABC subtype. Consistent with the results of Ki‐67 immunostaining, the Poly‐CC ABC tumors exhibited significant enrichment of gene sets related to cell proliferation and the cell cycle (Figure 4C). Interestingly, several signaling pathways, including IFN‐γ and IFN‐α responses, IL‐2/STAT5 and IL‐6/JAK/STAT3 signaling, inflammatory signaling, and complement activation, were more enriched in the Mono‐CC ABC tumors (Figure 4D). These findings suggest that within DLBCL tumors of the ABC subtype, Mono‐ and Poly‐CC tumors differ not only in their B‐cell differentiation states, but also in their tumor‐intrinsic biology.

### 3.5 Gene Mutation Analysis of Mono‐CC and Poly‐CC DLBCL

**FIGURE 5:**
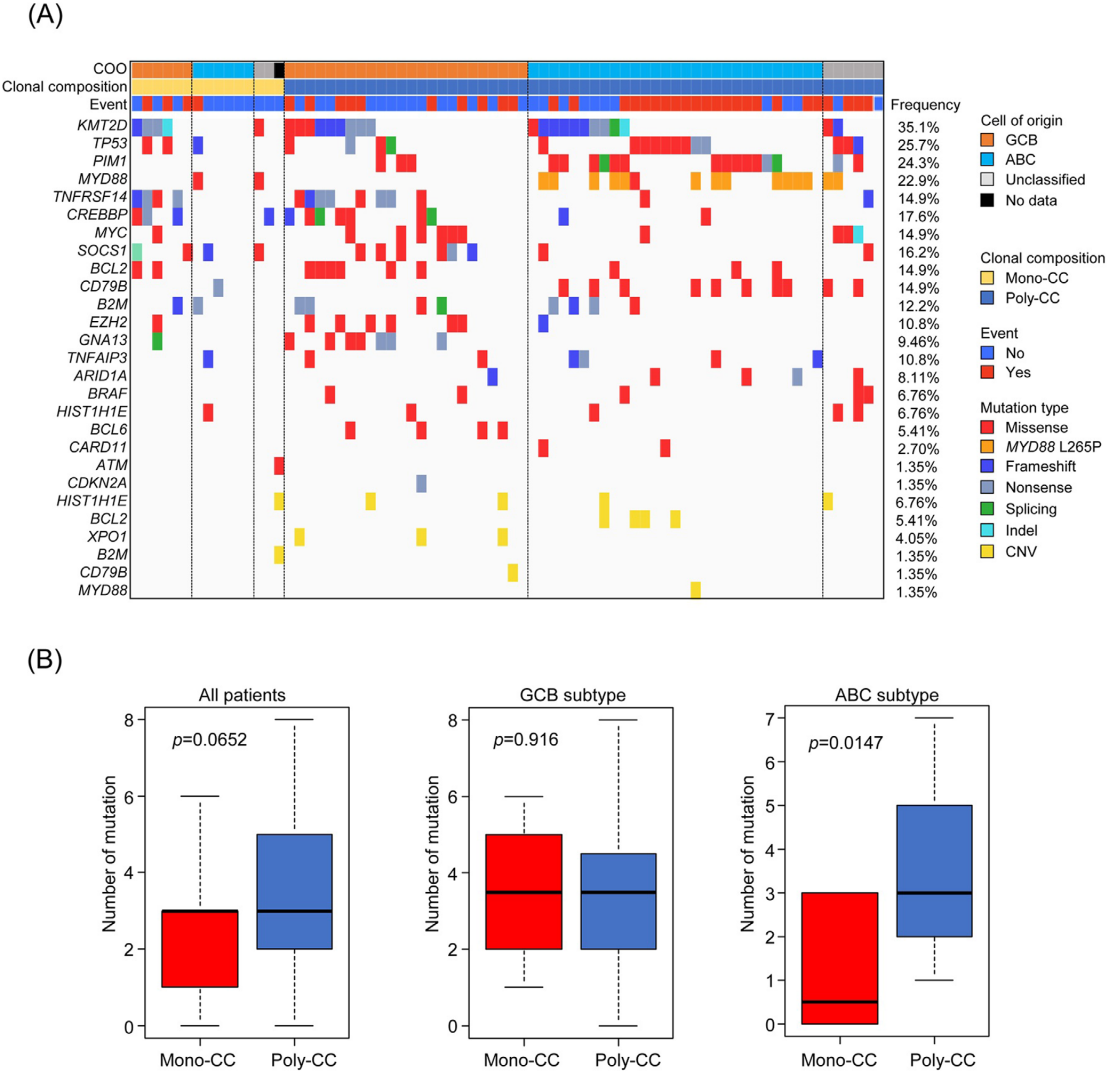
Relationships between intratumor heterogeneity and gene mutation status across DLBCL subtypes. (A) Genomic profile of DLBCL using Oncomine Lymphoma Panel. Oncoplot showing the distribution of recurrent mutations and copy number alterations. (B) Comparison of the number of mutations identified between Mono‐CC and Poly‐CC tumors classified into DLBCL subtypes according to the COO classification (Left, all cases; Middle, GCB subtype; Right, ABC subtype); *p* values were calculated using the Mann–Whitney U test.

Next, we evaluated the somatic mutation profiles of the tumors to explore the relationship between the CC number and the molecular background of DLBCL. This analysis was performed using genomic data of the 74 patients for whom the CC number could be calculated. For determining the somatic mutation profile, we used the Oncomine Lymphoma Panel containing 25 genes applicable to DLBCL (Figure 5A). The on‐target rate, defined as the percentage of sequencing data covering the targeted regions and reflecting sample quality, was 95.8% (range, 73.0%–98.1%). The median read depth was 4172 (range, 1657–5087). In the CC = 0 group, the on‐target rate was 94.3% (range, 73.0%–97.0%) and the median read depth was 4078 (range, 1657–4493). In the CC ≥ 1 group, the on‐target rate was 96.0% (range, 80.0%–98.1%) and the median read depth was 4249 (range, 2060–5087). Among all the specimens that could be successfully analyzed for gene mutations, the most frequently mutated genes were *KMT2D* (35.1%), followed by *TP53*, *PIM1*, *MYD88*, *TNFRSF14*, and *CREBBP*, all of which are commonly implicated in DLBCL. The mean number of mutations per sample was 3.20 (Figure 5A). We next compared the number of mutations between the Mono‐CC and Poly‐CC tumors. No significant difference was observed in the overall cohort (*p* = 0.0652; Figure 5B, left). Similarly, no significant difference was observed between the Mono‐CC and Poly‐CC tumors within the GCB subtype either (*p* = 0.916; Figure 5B, middle). Pathogenic mutations associated with the GCB subtype, including mutant *CREBBP*, *TNFRSF14*, and *KMT2D*, a well‐established driver gene in DLBCL, were frequently observed in both Mono‐CC and Poly‐CC GCB tumors (Figure 5A). In contrast, within the ABC subtype, the number of mutations per sample was significantly higher in the Poly‐CC tumors than in the Mono‐CC tumors (*p* = 0.0147; Figure 5B, right). Furthermore, ABC‐related driver mutations, including mutant *PIM1*, *MYD88* ^*L265P*^, and *CD79B*, were detected almost exclusively in the Poly‐CC ABC tumors, with few mutations found in the Mono‐CC ABC tumors (Figure 5A).

### 3.6 Analysis of the Tumor Microenvironment in Mono‐CC and Poly‐CC DLBCL

The tumor microenvironment (TME) is known to play a critical role in both the prognosis and biology of B‐cell lymphomas [39, 40, 41, 42]. Given the observed differences in the clinical outcomes and tumor‐intrinsic features between Mono‐CC and Poly‐CC tumors, we next investigated whether the composition of the TME also differed between these tumors. We applied the CIBERSORTx [32] tool to gene expression data to estimate the relative abundance of various immune cell populations. However, no significant differences were observed in the estimated fractions of any of the key components of the TME, including CD8^+^ T cells, CD4^+^ T cells, and macrophage subsets (M0, M1, M2) between the Mono‐CC and Poly‐CC tumors (Figure S6). This absence of difference in the composition of the TME was also observed when the GCB and ABC tumor subtypes were analyzed separately, suggesting that the characteristics of the TME do not exert a significant influence on the clonal complexity. To validate these results of analysis by CIBERSORTx, we additionally performed immunohistochemical staining of tumor specimens of the ABC subtype of DLBCL for CD3, CD4, CD8, CD68, and CD163. Consistent with the computational estimates, the results of immunohistochemistry also showed no significant differences in the composition of the TME between Mono‐CC and Poly‐CC ABC tumors (Figure S7). Taken together, the results of the aforementioned analyses of the TME suggest that the differences in the results of GSEA observed between the Mono‐CC and Poly‐CC tumors of the ABC subtype are more likely attributable to tumor‐intrinsic features than to variations in the tumor microenvironment.

## 4 Discussion

In this study, we applied a SNP‐array–based approach for copy number analysis to FFPE tumor specimens from newly diagnosed patients with advanced‐stage DLBCL (*n* = 74) and assessed the clonal complexity in the specimens by determining the CC number. Poly‐CC tumors, which accounted for 79.7% of the 74 cases included in the analysis, showed a trend toward a poorer prognosis as compared with Mono‐CC tumors. A noteworthy finding was that when the patients were classified by gene expression profiling according to the COO classification, the adverse prognostic impact of a high clonal complexity (Poly‐CC tumors) was statistically significant in the ABC subtype, but not the GCB subtype of DLBCL. Although several clinical factors may have contributed to the poor outcomes of Poly‐CC ABC tumors, the multivariable analysis suggests that both biological aggressiveness associated with clonal complexity and clinical factors such as treatment selection and extranodal disease likely played a role.

Most studies on DLBCL have focused on inter‐patient heterogeneity based on genetic alterations [19, 22]. However, a limited number of reports have also addressed the significance of ITH in this disease. Suguro et al. reported that 47% of DLBCL cases exhibited clonal heterogeneity as evidenced by array comparative genomic hybridization, which was associated with significantly poorer outcomes [24]. Similarly, Wang et al. classified approximately 50% of early‐stage DLBCL patients as having high ITH based on the mutant‐allele tumor heterogeneity (MATH) score derived from whole‐exome sequencing and reported that these patients were at a significantly higher risk of tumor progression [23]. The higher proportion of poly‐CC tumors observed in our study reflects the increased sensitivity of our CNV‐based approach (BAF/log2R) as well as the specific definition applied for clone calling. These methodological differences likely account for the discrepancy compared to previous reports. Taken together with our findings, these results suggest that DLBCL, like many solid tumors, contains multiple subclonal populations at diagnosis, which may influence the clinical outcomes.

A wide variety of computational methods have been developed to estimate ITH, including mutation‐based tools such as the MATH score [43], PyClone [44], and SciClone [45], copy number–based methods such as CNVkit [46] and FACETS [47], and repertoire‐based approaches like MiXCR [48]. While mutation‐based methods often provide higher sensitivity for detecting subclonal populations, they typically require high‐quality fresh or frozen tissue specimens and paired tumor/normal samples. In contrast, we used a model‐based metric specifically optimized for degraded DNA extracted from archival FFPE specimens, which are often the only accessible material in retrospective or routine clinical settings. In addition, it does not require paired tumor/normal samples or whole‐genome coverage, which significantly lowers the cost and technical barriers, enabling robust, quantitative, and reproducible assessment of clonal complexity. Given its successful application to both solid tumors and DLBCL, the approach may offer broad utility for characterizing ITH across a range of non‐Hodgkin lymphomas.

A key strength of our study lies in our comprehensive investigation of tumor‐intrinsic and extrinsic features underlying differences in clonal complexity. Firstly, GSEA revealed significant enrichment of cell cycle–related pathways, including E2F targets, MYC targets, and the G2M checkpoint, in Poly‐CC tumors. Consistent with this finding, immunohistochemical analysis showed significantly higher Ki‐67 positivity in Poly‐CC tumors. These findings suggest that increased proliferative activity might be a key biological feature associated with higher clonal complexity in DLBCL. This link between tumor cell proliferative activity and ITH is also corroborated by a previously reported study in lung adenocarcinoma, which reported a positive correlation between tumor Ki‐67 positivity and the burden of subclonal genetic alterations [49].

We also explored differences in the differentiation status of the malignant B cells using ECOtyper, a computational framework that infers cellular states based on gene expression signatures [31]. Interestingly, in the GCB‐DLBCL subgroup, no significant differences in the B‐cell differentiation states were observed between Mono‐CC and Poly‐CC tumors. In contrast, in the ABC‐DLBCL tumor group, Poly‐CC tumors showed more differentiated cell states (S4–S5), whereas the B cells in most Mono‐CC tumors retained earlier‐stage states. These findings were further reinforced by the results of ssGSEA, which showed upregulation of *IRF4*‐associated pathways, known regulators of B‐cell differentiation, in Poly‐CC tumors as compared with Mono‐CC tumors in the ABC‐DLBCL tumor group. Conversely, several signaling pathways, such as IFN‐α and IFN‐γ responses, inflammation, complement activation, IL‐6/JAK‐STAT3 signaling, and IL‐2/STAT5 signaling were markedly suppressed in Poly‐CC ABC tumors. While some of these pathways are traditionally considered to be immune‐related, tumor microenvironment analysis using both CIBERSORTx and immunohistochemistry showed no significant differences in the immune cell composition between Mono‐CC and Poly‐CC tumors. These findings suggest that these transcriptomic differences likely reflect tumor‐intrinsic properties rather than variations of the tumor microenvironment.

In parallel with the observed differentiation status, Poly‐CC tumors also harbored a significantly higher number of pathogenic mutations as compared with Mono‐CC tumors within the ABC‐DLBCL subgroup. Notably, ABC subtype–related driver mutations such as *PIM1*, *MYD88* ^*L265P*^, and *CD79B* were predominantly detected in Poly‐CC tumors, while they were largely absent in Mono‐CC tumors. These results suggest that tumors with higher clonal complexity not only adopt more differentiated cellular states but also accumulate a greater burden of genetic alterations, further reflecting evolutionary progression within the ABC subtype.

Recent advances in frontline therapy for DLBCL, such as substituting polatuzumab vedotin for vincristine in R‐CHOP, have demonstrated improved outcomes, particularly in the ABC subtype [50]. It is possible that the prognostic impact of clonal complexity, especially among ABC tumors, may be attenuated by the introduction of such targeted agents. Although all patients in our cohort received conventional R‐CHOP, future studies incorporating contemporary regimens will be needed to clarify how emerging therapies interact with tumor clonal architecture.

Several limitations of our study should be acknowledged. First, our assessment of ITH was based on copy number alterations without integration with other genomic or immunogenetic markers, such as B‐cell receptor clonality or somatic mutation profiling. Therefore, it remains unclear whether the observed ITH actually reflects evolutionary divergence from a common B‐cell precursor or the coexistence of distinct cellular origins. Second, we did not analyze paired samples at relapse, which limited our ability to determine which subclones might contribute to treatment resistance. Third, due to the limited sample size, larger cohort studies are needed to validate the prognostic implications of clonal complexity observed in this study. In addition, because our analysis relied on a single biopsy site, the full extent of intra‐tumoral heterogeneity may not have been captured. Detection using tumor‐derived DNA circulating in the blood has been extensively studied in solid tumors and more recently in DLBCL [5, 7, 51]. ctDNA‐based approaches enable detection of tumor‐specific genetic alterations and capture both intra‐ and intertumoral heterogeneity, offering a dynamic and minimally invasive strategy for disease monitoring. Future studies incorporating ctDNA analysis have the potential to provide new insights into clonal architecture and heterogeneity in DLBCL.

In conclusion, our study shows that newly diagnosed advanced‐stage DLBCL patients exhibit substantial ITH, with Poly‐CC ABC‐DLBCL cases potentially showing poorer outcomes. By analyzing the biology underlying ITH, we found that clonal complexity captures tumor‐intrinsic features and biological diversity in DLBCL, particularly in tumors of the ABC subtype. These findings offer new insights into DLBCL biology and may aid in advancing our understanding of the pathogenesis of DLBCL at the molecular level.

## Author Contributions

**Takahiro Haeno:** conceptualization (equal), formal analysis (lead), writing – original draft (lead), writing – review and editing (equal). **Kazuko Sakai:** conceptualization (equal), methodology (lead), writing – review and editing (equal). **Shuji Minamoto:** data curation (equal), investigation (equal), writing – review and editing (equal). **Daiki Nakatsu:** data curation (equal), investigation (equal), writing – review and editing (equal). **Marco A. De Velasco:** data curation (equal), investigation (equal), writing – review and editing (equal). **Shinya Rai:** supervision (equal), writing – review and editing (equal). **Hirokazu Tanaka:** data curation (equal), investigation (equal), writing – review and editing (equal). **Itaru Matsumura:** supervision (equal), writing – review and editing (equal). **Kazuto Nishio:** conceptualization (lead), writing – review and editing (equal).

## Ethics Statement

This study was conducted with the approval of the independent ethics committee of Kindai University Faculty of Medicine (R03‐283), adhering to the principles of the Declaration of Helsinki using.

## Consent

The requirement for written informed consent was waived by the committee, and information regarding the study was disclosed to the participants via an opt‐out method.

## Conflicts of Interest

Kazuko Sakai has received honoraria from Qiagen Inc., Takeda Pharmaceutical Co. Ltd., Nippon Kayaku Co. Ltd., and Life Technologies Japan Ltd., outside the submitted work. Itaru Matsumura reports grants from Chugai Pharmaceutical Co. Ltd., Kyowa Kirin Co. Ltd., Sumitomo Pharma, Asahi Kasei Pharma Corporation, Eisai Co. Ltd., Taiho Pharmaceutical Co. Ltd., AbbVie GK., and Otsuka Pharmaceutical Co. Ltd.; consulting fee from Otsuka Pharmaceutical Co. Ltd.; honoraria from Chugai, Novartis Pharma, Bristol‐Myers Squibb, Pfizer Japan, Otsuka Pharmaceutical Co. Ltd., AbbVie, Takeda Pharmaceutical Co. Ltd., Ono Pharmaceutical, SymBio Pharmaceuticals, Sanofi, Astellas Pharma, AstraZeneca, and Janssen Pharmaceutical, outside the submitted work. Kazuto Nishio reports grants from West Japan Oncology Group, Nichirei Biosciences Inc., Hitachi, Otsuka Pharmaceutical Co. Ltd., Thoracic Oncology Research Group, Eli Lilly Japan, Okayama University, Japan Breast Cancer Research Group, and Nippon Boehringer Ingelheim; consulting fee from SymBio Pharmaceuticals, Eli Lilly Japan, and Otsuka Pharmaceutical Co. Ltd.; honoraria from Chugai, MSD, Guardant Health, Daiichi Sankyo, Ono Pharmaceutical, Janssen Pharmaceutical, Novartis Pharma, Eli Lilly Japan, Invitae Japan, AstraZeneca, Nichirei, and Maruho, outside the submitted work. The remaining authors declare that they have no known competing financial interests or personal relationships that could have appeared to influence the work reported in this paper.

## Supporting information


**Figure S1:** Summary of this study.
**Figure S2:** Representative image of clonal composition analysis.
**Figure S3:** Survival analysis in patients with DLBCL.
**Figure S4:** Correlation between clonal composition number and Ki‐67 index, IPI score, and clinical stage in DLBCL subtypes.
**Figure S5:** Assessment of lymphoma ecotype in DLBCL using ECOtyper.
**Figure S6:** Immune cell composition of the tumors determined using CIBERSORTx.
**Figure S7:** Immunohistochemical staining.
**Table S1:** Gene set enrichment analysis (GSEA) results between the Mono‐CC and Poly‐CC tumors within the ABC subtype.
**Table S2:** Multivariate analysis for EFS in all patients.
**Table S3:** Multivariate analysis for EFS in patients with ABC subtype.

## Data Availability

Data generated or analyzed during this study are available from the corresponding author on reasonable request.
