# Supplementary material for: Clonal Complexity Defines Distinct Tumor‐Intrinsic Biology and Prognosis in Diffuse Large B‐Cell Lymphoma
Source: Cancer Med. 2026 Feb 2;15(2):e71597. doi: 10.1002/cam4.71597 (PMC12863995; doi:10.1002/cam4.71597)
Supplement: Supplementary file 1 — Figure S1: Summary of this study. Figure S2: Representative image of clonal composition analysis. Figure S3: Survival analysis in patients with DLBCL. Figure S4: Correlation between clonal composition number and Ki‐67 index, IPI score, and clinical stage in DLBCL subtypes. Figure S5: Assessment of lymphoma ecotype in DLBCL using ECOtyper. Figure S6: Immune cell composition of the tumors determined using CIBERSORTx. Figure S7: Immunohistochemical staining. Table S1: Gene set enrichment analysis (GSEA) results between the Mono‐CC and Poly‐CC tumors within the ABC subtype. Table S2: Multivariate analysis for EFS in all patients. Table S3: Multivariate analysis for EFS in patients with ABC subtype. [file CAM4-15-e71597-s001.docx]

**Clonal Complexity Defines Distinct Tumor-Intrinsic Biology and Prognosis in Diffuse Large B-cell Lymphoma**

Takahiro Haeno^1,2^, Kazuko Sakai^1,3^, Shuji Minamoto^1,2^, Daiki Nakatsu^1^, Marco A De Velasco^1^, Shinya Rai^2,4^, Hirokazu Tanaka^2^, Itaru Matsumura^2^, Kazuto Nishio^1,3^

^1^Kindai University Faculty of Medicine, Department of Genome Biology, Osaka-Sayama, Japan

^2^Kindai University Faculty of Medicine, Department of Hematology and Rheumatology, Osaka-Sayama, Japan

^3^Kindai University, Center for Genomics, Life Science Research Institute, Osaka-Sayama, Japan

^4^Centre for Lymphoid Cancer, BC Cancer, Vancouver, BC, Canada

**Corresponding author:** Kazuto Nishio, Department of Genome Biology, Kindai University Faculty of Medicine, 377-2 Ohno-higashi, Osaka-Sayama, Osaka 589-8511, Japan. Tel.: +81-72-366-0221. Fax: +81-72-360-5000. E-mail: knishio@med.kindai.ac.jp

This PDF file includes

1. Materials and Methods

2. Figure S1.

Summary of this study

3. Figure S2.

Representative image of clonal composition analysis

4. Figure S3.

Survival analysis in patients with DLBCL

5. Figure S4.

Correlation between clonal composition number and Ki-67 index, IPI score, and clinical stage in DLBCL subtypes.

6. Figure S5.

Assessment of lymphoma ecotype in DLBCL using ECOtyper.

7. Figure S6.

Immune cell composition of the tumors determined using CIBERSORTx

8. Figure S7.

Immunohistochemical staining

9.Table.S1

Gene set enrichment analysis (GSEA) results between the Mono-CC and Poly-CC tumors within the ABC subtype

10.Table.S2

Multivariate analysis for EFS in all patients

11.Table.S3

Multivariate analysis for EFS in patients with ABC subtype

12.Reference

**Supporting Materials and Methods**

**DNA and RNA isolation**

DNA and RNA were extracted from formalin-fixed paraffin embedded (FFPE) specimens using the AllPrep DNA/RNA FFPE kit (Qiagen), in accordance with the manufacturer’s instructions. The quality and quantity of the nucleic acids were verified using the NanoDrop 2000 device, PicoGreen dsDNA Reagent, and RiboGreen RNA reagent (all from Thermo Fisher Scientific).

**Clonal composition analysis**

Microarray-based comparative genomic hybridization assay for genome-wide estimation of copy number aberrations was performed with the use of the OncoScan FFPE Assay Kit (Thermo Fisher Scientific). Eighty ng of DNA was subjected to annealing with these molecular inversion probe (MIP) for 16 to 18 h, followed by enzyme digestion and two separate gap-fill reactions. The circular MIPs were then separately linearized for each gap fill with a cleavage enzyme and amplified by PCR. The PCR products were subjected to enzymatic cleavage and fragmentation, followed by hybridization for 16 to 18 h with two OncoScan arrays (one for each gap fill). The arrays were then stained and washed using GeneChip Fluidics Station 450 and loaded into GeneChip Scanner 3000 7G (Thermo Fisher Scientific). Array fluorescence intensity (CEL) files were generated with Affymetrix GeneChip Command Console (AGCC) software version 4.0, and the CEL files were converted to OSCHP files with OncoScan Console software 1.3.

Clonal composition analysis was performed using the B allele frequency (BAF) and log2 Ratio (log2R) information obtained from the OncoScan FFPE assay. A given copy number segment can be associated with a certain percentage of aberrant tumor cells (%AC), which is assumed to result from a single underlying event attributable to a single clone; in other words, copy number segments are associated with the same %AC and belong to the same clone. The clonal composition number was calculated by identifying the number of different %AC detected among the aberrant segments. The number of minor alleles (NOMA) at a heterozygous site can be defined as the BAF of a segment. Calculation of the clone composition number is performed using NOMA = 0, which represents a segment with 100% loss of heterozygosity and a normal copy number. If only one of such alleles is present, the BAF for such a segment will be zero. The Onco Clone Composition program provides analytical estimations of clonal composition using a clustering approach that clusters all segments with the same logR and BAF values and then combines clusters corresponding to the same %AC. A graphic representation of the aberrant segments and their association with the AC is shown in Supplementary Fig. 5

**Target DNA sequencing**

For DNA sequencing, 10 ng of DNA was subjected to multiplex PCR amplification with the use of the Oncomine Lymphoma Panel (Thermo Fisher Scientific). After multiplex PCR, Ion Xpress Barcode Adapters (Thermo Fisher Scientific) were ligated to the PCR products, which were then purified using Agencourt AMPure XP beads (Beckman Coulter). The purified libraries were pooled and then sequenced with the use of the Ion S5 XL system and Ion 550 Chip (all from Thermo Fisher Scientific). The DNA sequencing data were accessed through the Torrent Suite v.5.18 software (Thermo Fisher Scientific). Reads were aligned with the hg19 human reference genome and potential mutations were identified using Variant Caller version 5.18. Raw variant calls were manually checked with the integrative genomics viewer (IGV; Broad Institute). Germline mutations were excluded with the use of the Human Genetic Variation Database (<http://www.genome.med.kyoto-u.ac.jp/SnpDB>).

**Immunohistochemical staining and histopathological analysis**

FFPE specimens were sliced into 3- to 4-μm thick sections and subjected to hematoxylin-eosin staining. Immunohistochemical staining was performed using the following primary antibodies - anti-CD3 (2GV6, Roche), anti-CD4 (SP35, Roche), anti-CD8 (SP239, Roche), anti-CD68 (KP-1, Roche), anti-CD163 (MRQ-26, Roche), anti-Ki67 (30-9, Roche) - with the BenchMark Ultra platform (Ventana Medical System). The stained samples were digitally scanned using a Nanozoomer virtual slide scanner (Hamamatsu Photonics). The imaging analysis was performed using the HALO software (Indica Labs).

**Analysis of the tumor microenvironment**

The cells comprising the tumor microenvironment were estimated with the CIBERSORTx tool using normalized gene expression data (TPM). The LM 22 signature matrix was applied as reference for 22 immune cell subsets. Quantile normalization was not applied to the gene expression data, based on previous reports. [1, 2]

**Figure S1.**

**Summary of this study**

Oncoscan assay was performed on FFPE specimens prepared from 86 DLBCL patients, and the CC numbers could be calculated for 74 cases. RNA-seq gene expression analysis, gene mutation analysis by Oncomine Lymphoma panel, and immunohistochemical staining were performed on the 74 cases. RNA-seq analysis was feasible for 73 cases, while Oncomine Lymphoma panel analysis was feasible for all 74 cases. Using these data, survival analysis, cell of origin classification, ECOtyper, GSEA, ssGSEA, and CIBERSORTx were performed.


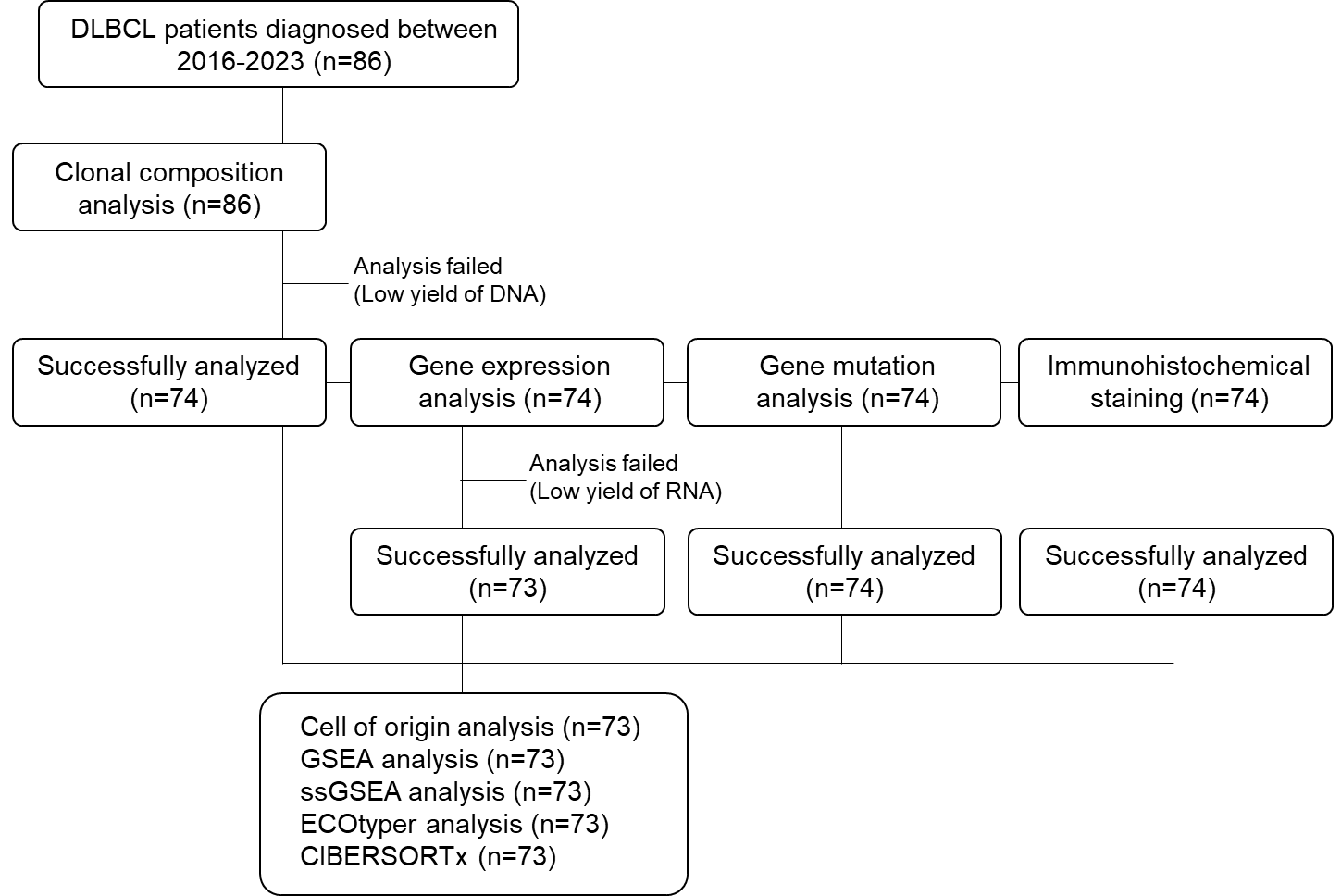


**Figure S2.**

**Representative image of clonal composition analysis**

The OncoClone Composition program plots in each of the panels on the left were derived from DLBCL samples with a CC number of 1 (A), 2 (B), and 3 (C). The arrows indicate the clonal composition. The x-axis depicts the BAF, and the y-axis the log2R for copy number. The panels on the right show a graphic representation of the log2R and BAF for each aberrant segment indicated by the numbered yellow circles aligned with the line of the same abnormal cell percentage. Each clear circle corresponds to one segment in the cancer sample. All selected clones with their associated %AC were selected and the corresponding segments turned yellow. Two representative cases (DL035 and DL072) with CC = 1 showing evidence of potential subclonal structure (D, E). Arrows indicate the major clone and a minor subclone. The proportion of markers assigned to each clone is shown in the corresponding panel.


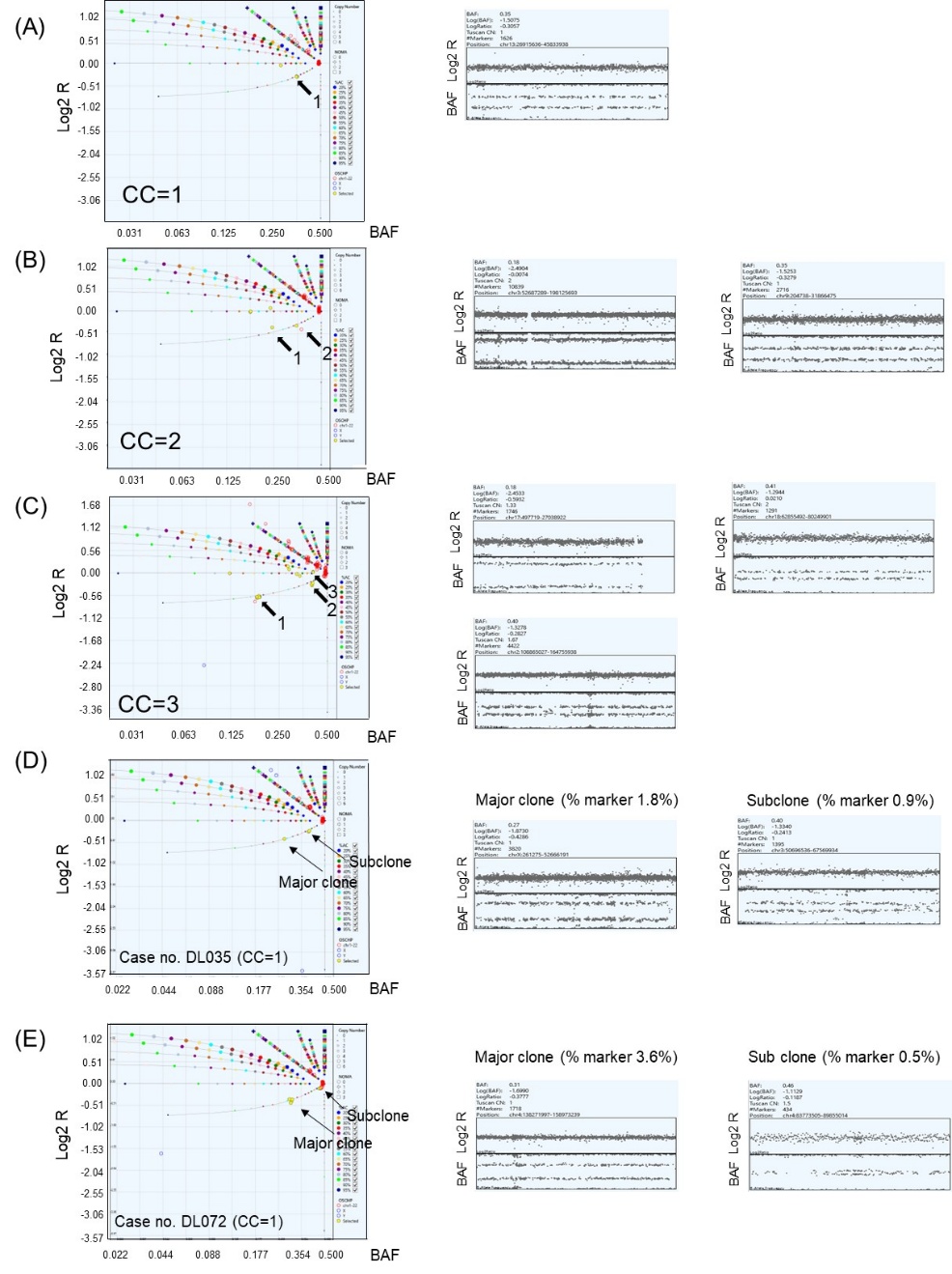


**Figure S3.**

**Survival analysis in patients with DLBCL**

(A) Comparison of the event-free survival between patients with the GCB and ABC subtypes of DLBCL (n=65). (B) Comparison of the overall survival between patients with the GCB and ABC subtypes of DLBCL (n=65). (C) Overall survival rates of patients with Mono-CC and Poly-CC tumors in the 74 cases of DLBCL. *p*-values calculated by the log rank test are shown in the graphs.


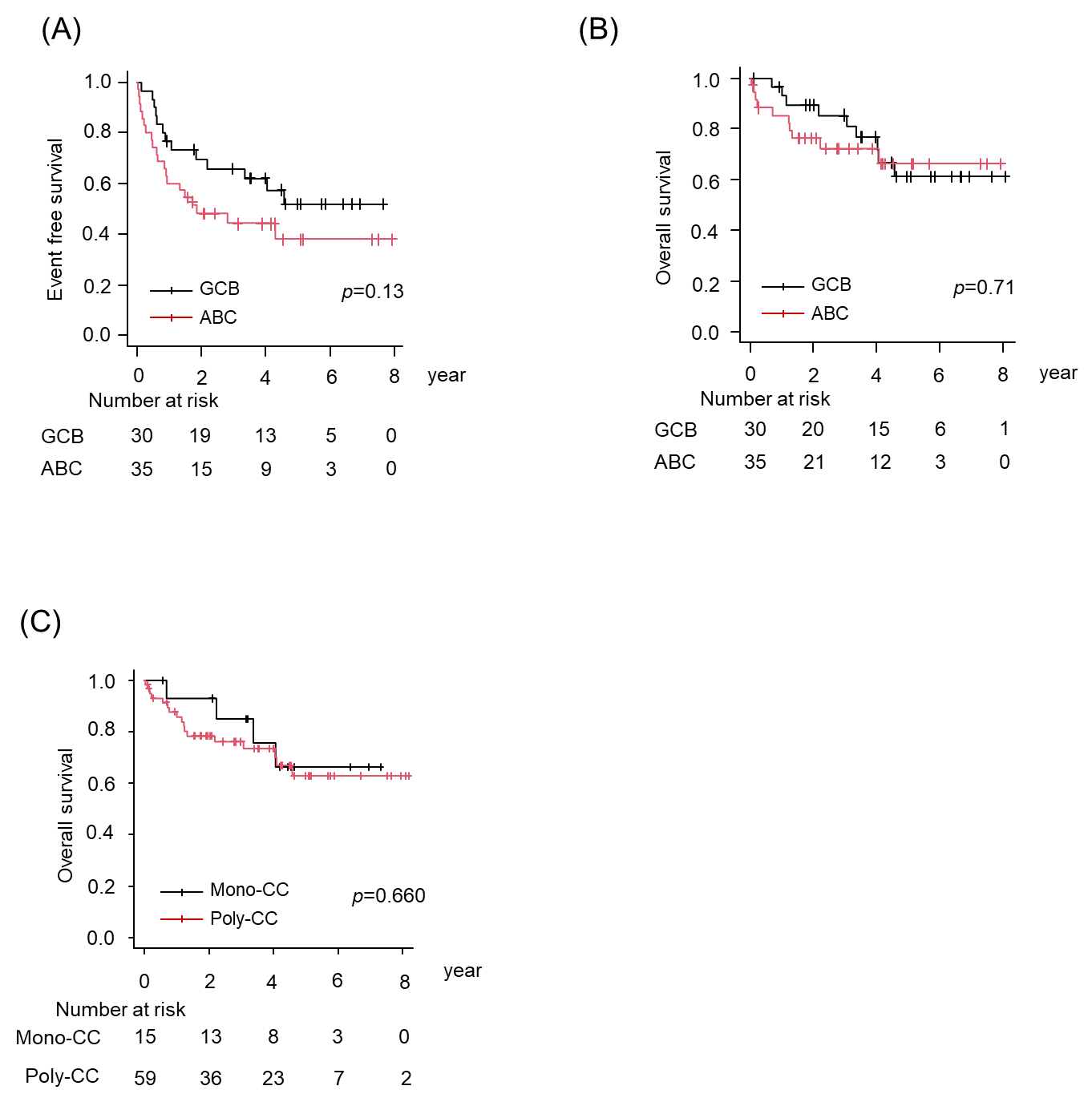


**Figure S4.**

**Correlation between clonal composition number and Ki-67 index, IPI score, and clinical stage in DLBCL subtypes.**

Scatter plots and box plots show the relationships between the clonal composition number (actual CC number) and Ki-67 index, IPI score, and clinical stage in all patients, the GCB subtype, and the ABC subtype. (A–C) Correlation between CC number and Ki-67 index (%Ki-67–positive cells). (D–F) Correlation between CC number and IPI score. (G–I) Comparison of CC number according to clinical stage (Stage 3 vs. Stage 4). Correlation coefficients (r), 95% confidence intervals (CI), and *p* values in panels (A–F) were calculated using Pearson’s correlation test, while differences between stages in panels (G–I) were evaluated using the Mann–Whitney *U* test.


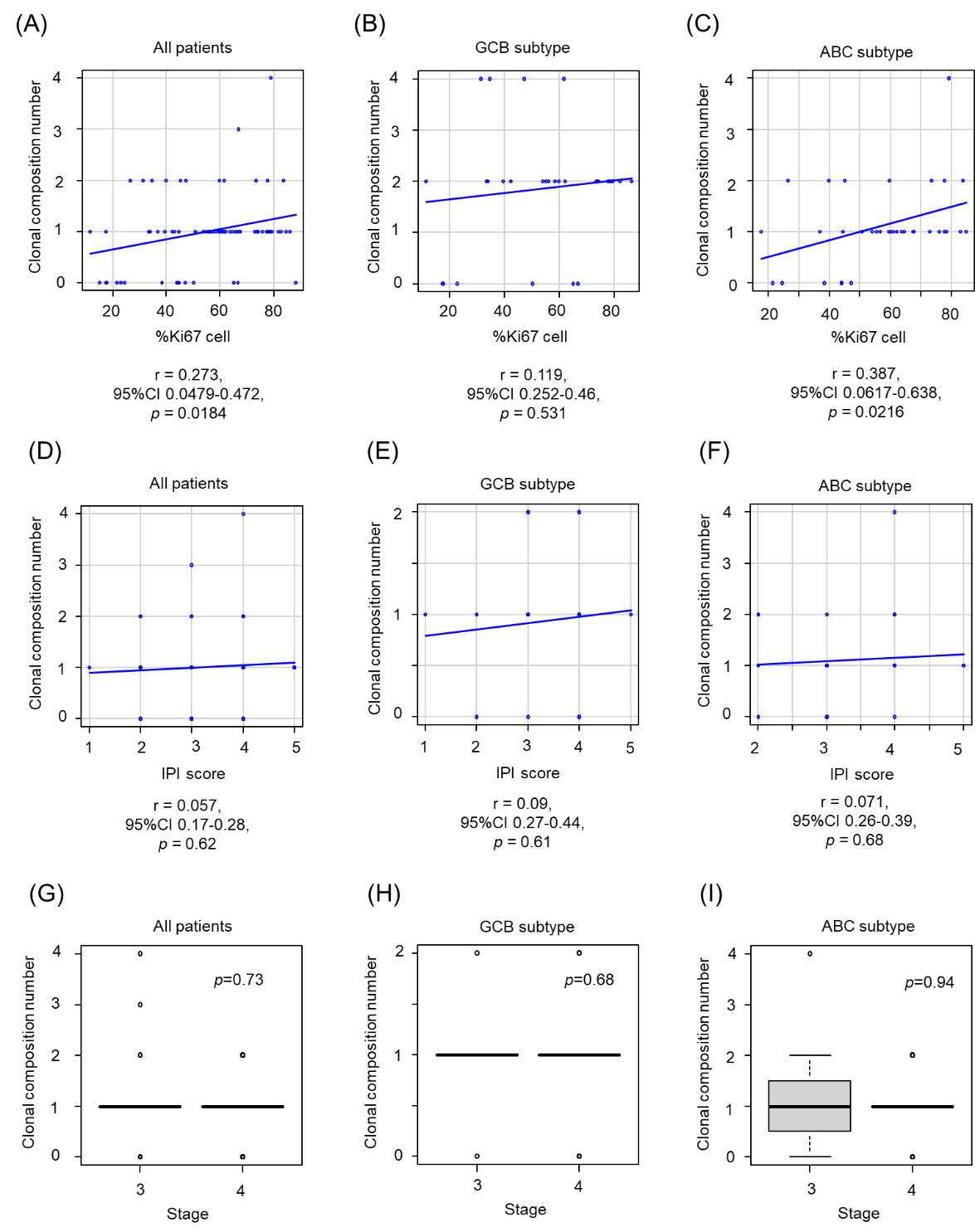


**Figure S5.**

**Assessment of lymphoma ecotype in DLBCL using ECOtyper**

Stacked bar plots show the relative proportions of EcoTyper-inferred Lymphoma Ecotypes (LE1–LE9) in individual cases classified as GCB (blue) or ABC (orange) subtypes. Gray bars indicate unclassified samples. Black bars indicate samples with no available cell-of-origin data.

NA, not assigned

**
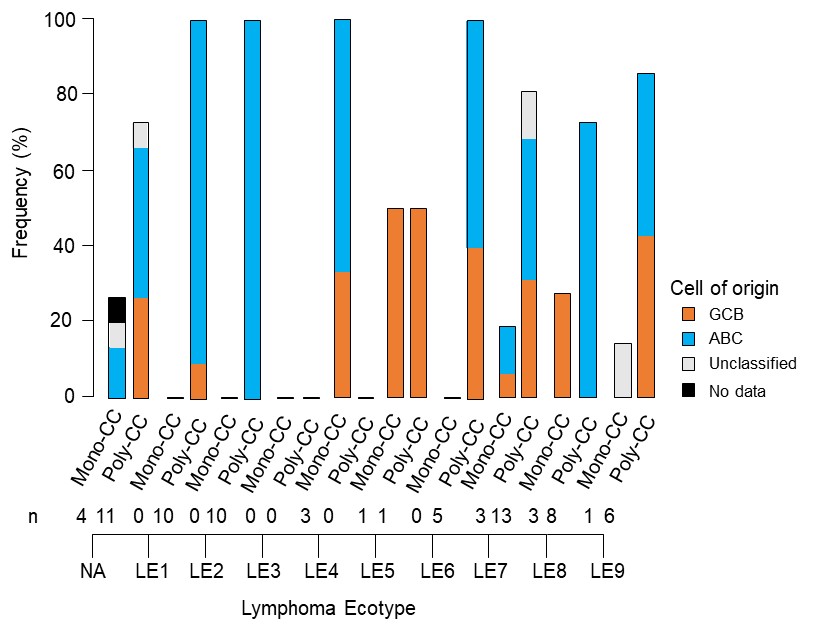
**

**Figure S6.**

**Immune cell composition of the tumors determined using CIBERSORTx**

Box-plots depicting the comparative distributions of CD8^+^ T-cells, CD4^+^ T-cells, memory CD4^+^ T-cells, and macrophages (M0, M1, M2) between Mono-CC and Poly-CC tumors. This comparison was performed in all patients, including those with the GCB subtype and ABC subtype of DLBCL; *p*-values were calculated using the Mann-Whitney U test.


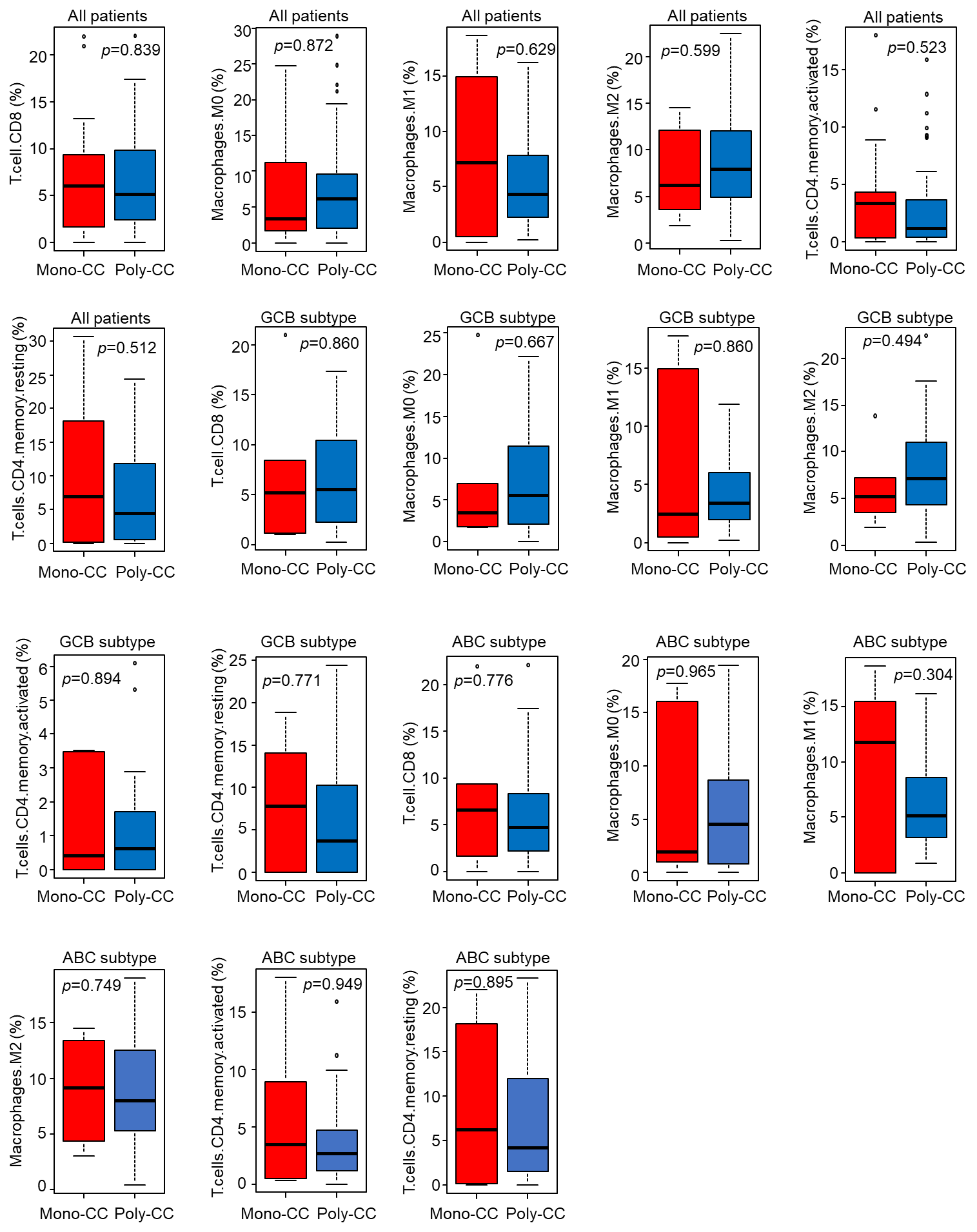


**Figure S7.**

**Immunohistochemical staining**

Comparison of the number of tumor cells showing positive IHC staining for CD3^+^, CD4^+^, CD8^+^, CD68^+^, and CD163^+^ between the Mono-CC and Poly-CC tumors in DLBCL patients with the ABC subtype; *p*-values were calculated using the Mann-Whitney U test.


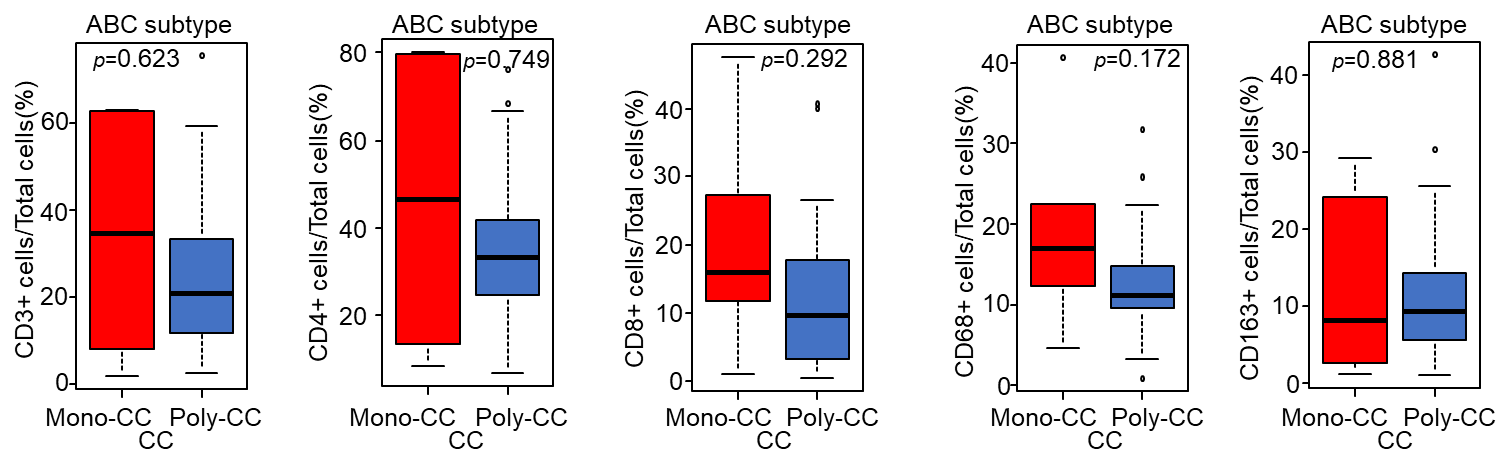


**Table S1.**

**Gene set enrichment analysis (GSEA) results between the Mono-CC and Poly-CC tumors within the ABC subtype**

|  | MSigDB hallmark pathway | NES | Nom *p* value | FDR *q* value |
| --- | --- | --- | --- | --- |
| Upregulated signatures | E2F targets | 1.68 | 0 | <0.01 |
|  | KRAS signaling DN | 1.57 | <0.01 | 0.0140 |
|  | MYC targets v2 | 1.57 | 0.0120 | 0.0120 |
|  | G2M checkpoint | 1.33 | 0.0210 | 0.150 |
| Downregulated signatures | Interferon γ response | -3.26 | 0 | 0 |
|  | Interferon α response | -3.23 | 0 | 0 |
|  | Inflammatory response | -2.80 | 0 | 0 |
|  | Complement | -2.58 | 0 | 0 |
|  | IL6/JAK/STAT3 signaling | -2.51 | 0 | 0 |
|  | Allograft rejection | -2.46 | 0 | 0 |
|  | IL2/STAT5 signaling | -2.29 | 0 | 0 |
|  | TNFα signaling via NFκB | -2.24 | 0 | 0 |
|  | Hypoxia | -2.08 | 0 | 0 |
|  | Epithelial mesenchymal transition | -1.78 | 0 | <0.01 |
|  | Apoptosis | -1.72 | 0 | <0.01 |
|  | Coagulation | -1.64 | 0 | <0.01 |
|  | mTORC1 signaling | -1.58 | 0 | <0.01 |
|  | UV response up | -1.56 | 0 | 0.0100 |
|  | Protein secretion | -1.60 | 0 | <0.01 |
|  | KRAS signaling up | -1.54 | 0 | 0.0120 |
|  | Oxidative phosphorylation | -1.52 | 0 | 0.0130 |
|  | Adipogenesis | -1.44 | 0 | 0.0240 |
|  | UV response DN | -1.35 | 0.0200 | 0.0440 |
|  | p53 pathway | -1.26 | 0.0240 | 0.0790 |
|  | Cholesterol homeostasis | -1.50 | 0.0090 | 0.0150 |
|  | Xenobiotic metabolism | -1.35 | 0 | 0.0430 |
|  | Androgen response | -1.49 | 0.0260 | 0.0150 |

NES; Normalized enrichment score, Nom *p* value; Nominal *p* value, FDR q value; False discovery rate *q* value

**Table S2.** **Multivariate analysis for EFS in all patients**

| Factor | Group | n | EFS | |
| --- | --- | --- | --- | --- |
|  |  |  | multivariate | |
|  |  |  | HR | *p*-value |
| CC number | Mono-CC  Poly-CC | 15  59 | 2.64(0.93-7.47) | 0.06 |
| 1st therapy | R-CHOP  RTHPCOP  R-CVP | 52  21  1 | 1.33(0.68-2.62) | 0.39 |
| Number of extranodal sites | 0-1  ≥2 | 40  34 | 1.48(0.77-2.84) | 0.23 |

**Table S3.** **Multivariate analysis for EFS in patients with ABC subtype**

| Factor | Group | n | EFS | |
| --- | --- | --- | --- | --- |
|  |  |  | multivariate | |
|  |  |  | HR | *p*-value |
| CC number | Mono-CC  Poly-CC | 6  29 | 5.994(0.77-46.6) | 0.087 |
| 1st therapy | R-CHOP  R-THPCOP  R-CVP | 21  13  1 | 2.41(0.93-6.23) | 0.067 |
| Number of extranodal sites | 0-1  ≥2 | 20  15 | 2.45(0.90-6.67) | 0.078 |

1. A. M. Newman, A. J. Gentles, C. L. Liu, M. Diehn, A. A. Alizadeh, "Data normalization considerations for digital tumor dissection," *Genome Biol* 18, no. 1 (2017): 128, <https://doi.org/10.1186/s13059-017-1257-4>.

2. A. M. Newman, C. B. Steen, C. L. Liu, et al., "Determining cell type abundance and expression from bulk tissues with digital cytometry," *Nat Biotechnol* 37, no. 7 (2019): 773–782, <https://doi.org/10.1038/s41587-019-0114-2>.
